# The association of iron deficiency anemia and perioperative complications following revision total knee arthroplasty

**DOI:** 10.1186/s42836-022-00129-4

**Published:** 2022-07-27

**Authors:** Stefan Hamaway, Bana Hadid, Rushabh M. Vakharia, Mitchell K. Ng, Adam M. Gordon, Martin W. Roche, Afshin E. Razi

**Affiliations:** 1 Department of Orthopaedic Surgery, Maimonides Medical Center, 927 49th Street, Brooklyn, NY 11219 USA; 2 College of Medicine, State University of New York Downstate, Brooklyn, NY USA; 3 Department of Orthopaedic Surgery, Hospital for Special Surgery, West Palm Beach, FL USA

**Keywords:** Revision Total Knee Arthroplasty, Iron Deficiency Anemia, Costs, Length of Stay, Complications

## Abstract

**Background:**

Recent studies show an increase in the prevalence of iron deficiency anemia (IDA) worldwide and a concomitant rise in the number of revision total knee arthroplasty (RTKA). The literature evaluating the association between IDA and perioperative outcomes following RTKA are limited. Therefore, the purpose of this study was to determine whether IDA patients undergoing RTKA have higher rates of (1) in-hospital lengths of stay (LOS), (2) complications; and (3) costs.

**Methods:**

Using International Classification of Disease, Ninth Revision (ICD-9) and Current Procedural Terminology (CPT), a retrospective query was performed from January 1^st^, 2005 to March 31^st^, 2014. The inclusion criteria consisted of those patients who have IDA undergoing RTKA. Study group patients were 1:5 ratio matched to a comparison cohort by age, sex, and various comorbidities: coronary artery disease, chronic obstructive pulmonary disease, diabetes mellitus, hyperlipidemia, hypertension, obesity, and tobacco use, yielding a total of 106,534 patients within the study (*n* = 17,784) and control (*n* = 88,750) cohorts. Outcomes assessed included: in-hospital LOS, costs of care, and medical complications. Multivariate Logistic regression analyses were used to calculate the odds-ratios (OR) and respective 95% confidence intervals (95%CI). Welch’s *t-*tests were used to compare in-hospital LOS and costs of care. Following Bonferroni-correction, a *P-*value less than 0.001 was considered statistically significant.

**Results:**

IDA patients undergoing RTKA were found to have significantly higher in-hospital LOS (4-days *vs*. 3-days, *P* < 0.0001). Additionally, IDA patients were found to have significantly higher odds (OR) of medical complications (OR: 5.29, *P* < 0.0001) such as: pneumonia (OR: 6.86, *P* < 0.0001), respiratory failures (OR: 5.95, *P* < 0.0001), myocardial infarctions (OR: 4.31, *P* < 0.0001) and other complications. Furthermore, IDA patients incurred significantly higher day of surgery ($16,976.01 *vs*. $14,515.81, *P* < 0.0001) and 90-day costs ($22,548.71 *vs*. $16,819.15, *P* < 0.0001).

**Conclusion:**

The study demonstrated IDA patients undergoing RTKA have higher rates of in-hospital LOS, costs of care, and medical complications. Orthopedic surgeons and other healthcare professionals can use this information to adequately educate these patients of the potential complications following their procedure.

**Supplementary Information:**

The online version contains supplementary material available at 10.1186/s42836-022-00129-4.

## Background

Studies have shown that preoperative anemia to be a prevalent comorbidity in patients undergoing primary elective total joint arthroplasty (TJA), with the frequency of the condition ranging from 21 to 35%, depending on the study being referenced [1–3]. One subset of anemic disorders is iron deficiency anemia (IDA), which is the leading cause of anemia worldwide, with one study citing that IDA contributed nearly 50% to all the anemic disorders [4]. While the rate of IDA is low in the United States, approximately 10 million people are iron-deficient, with risk factors for the condition including blood loss, especially in the elderly, insufficient iron intake, and malabsorptive conditions, such as celiac disease [5]. The societal and economic consequences of the condition are vast, leading to decreased work capacity and increased healthcare expenditures [4, 6]. Moreover, studies have demonstrated that IDA was associated with the development of adverse events following primary TJA [2, 7, 8].

In a retrospective study utilizing their own institution’s database, Viola *et al.* found that preoperative anemia following primary TJA was associated with higher rates of cardiovascular-related complications [7]. Chamieh *et al.,* utilizing an administrative database, showed that similar results that preoperative anemia in primary total knee arthroplasty (TKA) patients were associated with higher rates of pulmonary and renal-related complications [2]. In a more recent investigation, Mathew *et al.* illustrated that IDA increased the frequency and odds of complications with the episode of care interval following primary TKA [8]. While the aforementioned studies evaluated the anemia in the setting of primary TJA, currently, studies with a large sample size of patients utilizing multivariate analysis to determine the association of IDA in the setting of revision total knee arthroplasty (RTKA) have not been thoroughly elucidated. Understanding the relationship between IDA and RTKA will be essential to not only improving post-surgical patient care and potentially to mitigating the increased healthcare costs associated with this condition, but will serve to enhance the current literature on a comorbid condition with increasing prevalence worldwide [4, 9]. With the parallel rise of RTKA procedures being performed worldwide, studies evaluating the impact of IDA on RTKA outcomes are warranted [10].

As such, the aim of this study was to query a nationwide administrative claims database to identify IDA patients undergoing RTKA and to compare outcomes against a matched-cohort population. Specifically, this retrospective analysis examined whether IDA patients undergoing RTKA were correlated with higher rates of: (1) in-hospital lengths of stay (LOS); (2) healthcare expenditures; and (3) medical complications.

## Methods

In order to investigate adverse outcomes of IDA patients undergoing RTKA procedures, a retrospective level III case-control query from January 1^st^, 2005 to March 31^st^, 2014 using the PearlDiver Patient Records Database (http://www.pearldiverinc.com, Colorado Springs, Colorado) platform was performed. The supercomputer contains deidentified patient information from a private payor and the Medicare administrative claims and consists of over 100 million patients. Due to a myriad of research variables available, the for-fee based platform has been used extensively by investigators. Information is queried using diagnostic and reimbursement billing terminology in the form of International Classification of Disease, the Ninth Revision (ICD-9) and Current Procedural Terminology (CPT). Since the current study does not contain patient information, the current investigation was deemed exempt from our Institutional Review Board (IRB) review process.

The database was initially queried by author S.H. to identify all patients who underwent revision TKA using ICD-9 procedural codes 84.57, 81.55, 00.80 to 00.84. The database was then queried for all patients with IDA using ICD-9 diagnostic code 280.1 to 280.9. These codes were chosen as they have been used in previous investigations to define RTKA procedures and diagnosis of IDA [8, 11]. To ensure that patients queried within the database were counted only once, the “FIRST_INSTANCE” command syntax was utilized to mitigate overestimation on the association of IDA on the outcomes assessed. RTKA patients who have IDA at the time of their revision procedure represented the study cohort, whereas patients without IDA served as the control cohort. Ratio matching at a 1:5 proportion was utilized to minimize the effects of confounding and increase the overall sample size of the study [12]. Both cohorts were matched by the following matching parameters within the PearlDiver platform: age, sex, and the following medical comorbidities associated with IDA, including coronary artery disease, chronic obstructive pulmonary disease (COPD), diabetes mellitus, hyperlipidemia, hypertension, obesity, and tobacco use [13–16].

Primary variables to compare and assess between the study and matched-cohort population included: in-hospital lengths of stay (LOS), day of surgery and total global 90-day episode of care costs, and 90-day medical complications. Reimbursements were used as a substitute for costs of care, as it is a more accurate representation of what providers are paid from the insurance companies. Economic data were aggregated by the database tabulating all of the costs which patients in the cohorts may encounter on the day of their procedure to the time interval period of the investigator’s interest, in this case, 90-days following the index procedure. Information tabulated in the economic data can include, but not limited to: prescription refills, office visits, diagnostic tests pertaining to the index procedure, and other metrics [17–19]. Medical complications analyzed included: acute kidney injury, cerebrovascular accidents, deep vein thromboses, ileus, myocardial infarction, pneumonia, pulmonary emboli, respiratory failure, surgical site infection, urinary tract infection, and venous thromboemboli. Medical complications were defined using the ICD-9 codes found in the Supplementary Table 1.

## Data analyses

Baseline demographics of the matched cohorts were analyzed by Pearson’s Chi-Square Analyses. Welch’s *t-*test was used to compare in-hospital LOS and healthcare expenditures between the cohorts. Multivariate Logistic regression analyses were used to determine the association of IDA with postoperative complications within the episode of care period. The regression model calculated odds-ratios (OR) and their respective 95% confidence intervals (95%CI) adjusted for age, sex, geographic region, and Elixhauser-Comorbidity Index (ECI). While the Charlson-Comorbidity Index (CCI) has historically been used for observational studies, the current investigation relied on ECI as an adjusting covariate as ECI has been shown not to be only superior to CCI, but also to contain nearly twice as many comorbid conditions, ensuring additional covariate adjustment within the data analyses [20, 21]. Due to the ease of finding statistical differences with large administrative claims databases, a Bonferroni-correction was performed to reduce the probability of a type I error, and an alpha-value less than 0.002 was utilized as the threshold to determine statistical significance in the study. This value was obtained by taking 0.05 and dividing it by the total number of dependent variables analyzed in the current investigation (*n* = 17), as done in previously published studies using administrative claims registries [22–25]. Statistical analyses were performed using R (R, Foundation for Computational Statistics, Vienna, Austria).

## Results

The query yielded a total of 106,534 patients in the study (17,784) and control (88,750) cohorts. Matching was successful as there was no statistical difference between the matched cohorts (Table 1).

**Table 1:** Comparison of demographics of patients with iron deficiency anemia and matched-controls undergoing revision total knee arthroplasty. COPD = Chronic Obstructive Pulmonary Disease

Demographics	Iron Deficiency Anemia		Matched- Cohort		
	*n*	%	*n*	%	*P-*value^a^
Age (Years)					0.99
< 64	3,396	19.10	16,936	19.08	
65—69	5,160	29.01	25,777	29.04	
70—74	3,827	21.52	19,109	21.53	
75—79	2,896	16.28	14,432	16.26	
80—84	1,707	9.60	8,519	9.60	
85 <	798	4.49	3,977	4.48	
Sex					0.99
Female	9,988	56.16	49,851	56.17	
Male	7,796	43.84	38,899	43.83	
Comorbidities					
Coronary Artery Disease	6,210	34.92	30,972	34.90	0.96
COPD	278	1.56	1,283	1.45	0.99
Diabetes Mellitus	6,355	35.73	31,701	35.72	0.97
Hyperlipidemia	11,495	64.64	57,374	64.65	0.98
Hypertension	15,407	86.63	76,903	86.65	0.96
Obesity	2,234	12.56	11,097	12.50	0.84
Tobacco	2,735	15.38	14,577	16.42	0.79

^a^ Assessed by Pearson’s Chi-Square Analyses

### In-hospital length of stay & healthcare expenditures

**Fig. 1:**
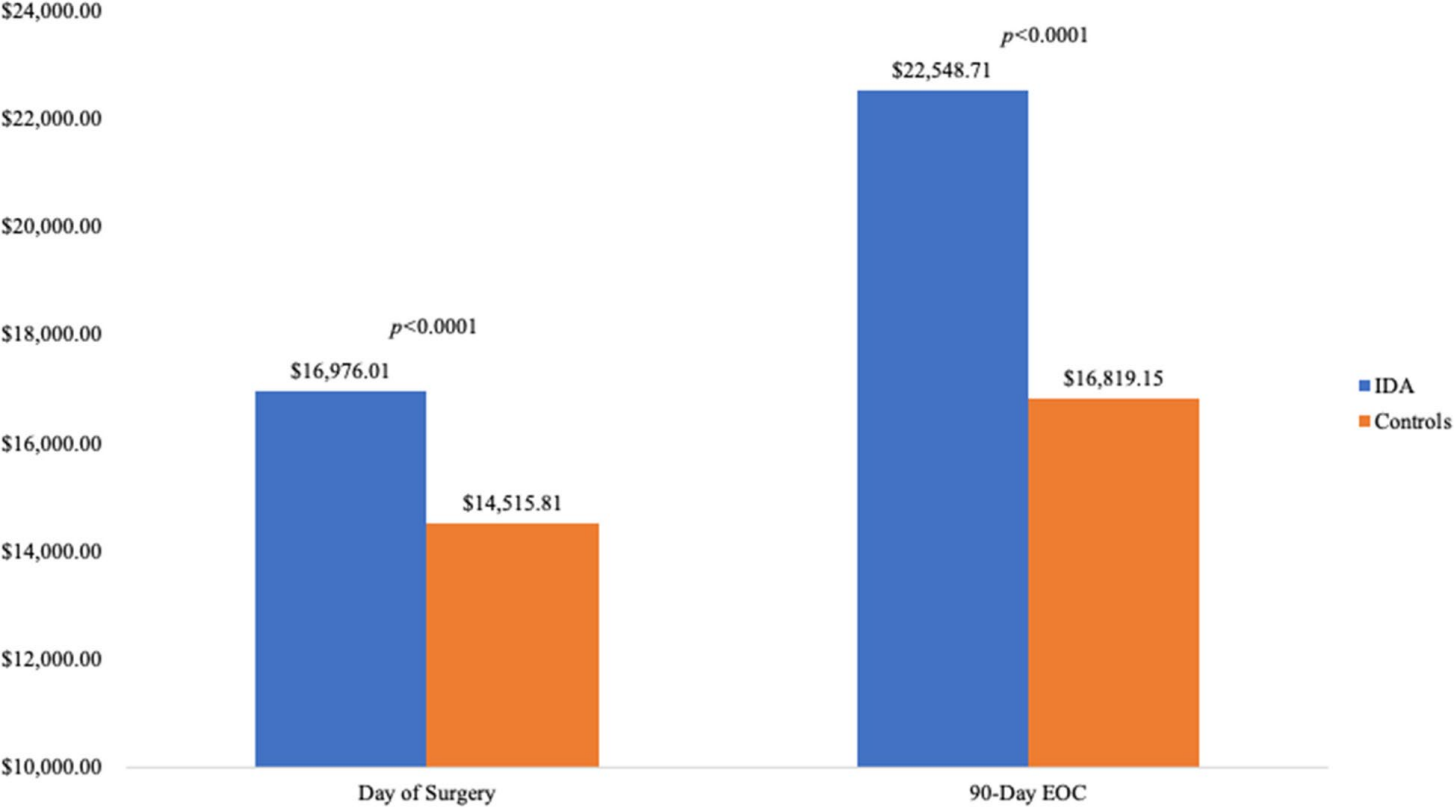
Comparison of day of surgery and total global 90-day episode of care costs amongst iron deficiency anemia patients undergoing revision total knee arthroplasty. EOC = Episode of Care; IDA = Iron Deficiency Anemia

The study found that patients with IDA undergoing RTKA had significantly longer in-hospital LOS (4-days *vs*. 3-days, *P* < 0.0001), compared to patients without IDA undergoing the same procedure. Female IDA patients (4-days) had longer in-hospital LOS compared to their male counterparts (3-days). When analyzing healthcare expenditures between the two cohorts, IDA patients undergoing RTKA incurred significantly higher day of surgery ($16,976.01 *vs*. $14,515.81, *P* < 0.0001) costs compared to their non-IDA counterparts. Similarly, study group patients were found to have significantly higher 90-day episode of care costs ($22,548.71 *vs*. $16,819.15, *P* < 0.0001) compared to the matched-control population (Fig. 1).

### Postoperative complications

IDA patients were also found to have significantly higher incidence and odds of medical complications (69.46% *vs*. 9.54%; OR: 5.29, 95%CI: 5.07 – 5.53, *P* < 0.0001) compared to their non-IDA counterparts. Specifically, the study found that IDA patients had significantly higher frequency and odds of developing acute kidney injuries (11.44% *vs*. 0.88%; OR: 7.06, 95%CI: 6.45 – 7.72, *P* < 0.0001), pneumonia (8.27% *vs*. 0.66%; OR: 6.86, 95%CI: 6.20 – 7.62, *P* < 0.0001), respiratory failures (7.18% *vs*. 0.61%; OR: 5.95, 95%CI: 5.33 – 6.64, *P* < 0.0001), ileus episodes (1.59% *vs*. 0.14%; OR: 5.62, 95%CI: 4.48 – 7.08, *P* < 0.0001), urinary tract infections (18.18% *vs*. 2.65%; OR: 5.21, 95%CI: 4.90 – 5.54, *P* < 0.0001), myocardial infarctions (1.95% *vs*. 0.22%; OR: 4.31, 95%CI: 3.55 – 5.24, *P* < 0.0001), cerebrovascular accidents (4.35% *vs*. 0.60%; OR: 4.03, 95%CI: 3.57 – 4.56, *P* < 0.0001), deep vein thromboses (4.07% *vs*. 0.89%; OR: 3.34, 95%CI: 2.99 – 3.73*, P* < 0.0001), surgical site infections (6.35% *vs*. 1.58%; OR: 3.22, 95%CI: 2.95 – 3.54, *P* < 0.0001), venous thromboemboli (4.61% *vs*. 1.02%; OR: 3.22, 95%CI: 2.90 – 3.57, *P* < 0.0001), and pulmonary emboli (1.47% *vs*. 0.28%; OR: 2.83, 95%CI: 2.33 – 3.43, *P* < 0.0001) (Table 2).

**Table 2:** Differences in ninety-day medical complications following revision total knee arthroplasty in iron deficiency anemia patients and matched-controls

Medical Complications Assessed	IDA (%)	Controls (%)	OR	95%CI	*P-*value^a^
Acute Kidney Injuries	11.44	0.88	7.06	6.45 – 7.72	< 0.0001
Pneumoniae	8.27	0.66	6.86	6.20 – 7.62	< 0.0001
Respiratory Failures	7.18	0.61	5.95	5.33 – 6.64	< 0.0001
Ileus Episodes	1.59	0.14	5.62	4.48 – 7.08	< 0.0001
Urinary Tract Infections	18.18	2.65	5.21	4.90 – 5.54	< 0.0001
Myocardial Infarctions	1.95	0.22	4.31	3.55 – 5.24	< 0.0001
Cerebrovascular Accidents	4.35	0.60	4.03	3.57 – 4.56	< 0.0001
Deep Vein Thromboses	4.07	0.89	3.34	2.99 – 3.73	< 0.0001
Surgical Site Infections	6.35	1.58	3.22	2.95 – 3.54	< 0.0001
Venous Thromboemboli	4.61	1.02	3.22	2.90 – 3.57	< 0.0001
Pulmonary Emboli	1.47	0.28	2.83	2.33 – 3.43	< 0.0001
Total Medical Complications	69.46	9.54	5.29	5.07 – 5.53	< 0.0001

*IDA* Iron Deficiency Anemia, *OR* Odds-Ratio, *95%CI* 95% Confidence Interval

^a^ Adjusted for Age, Sex, Geographic Region, and Elixhauser-Comorbidity Index

## Discussion

In the United States, there is an increase in the rates of primary TKA disproportionately affecting younger patients, that, when combined with the increased longevity of the population, will increase the demand for RTKA. Studies have conjectured that by 2030, there will be a 600% increase in the United States, amounting to over 250,000 procedures [10]. Concurrently, IDA is an important comorbidity that is associated with poor surgical outcomes, with as many as 21% to 35% of patients undergoing elective primary and revision TKA having been diagnosed as being anemic [7]. With the conjectured increase in the number of RTKA procedures worldwide within the next decade and high prevalence of IDA, the purpose of this investigation was to evaluate the association between IDA and patients undergoing RTKA. After adjusting for age, sex, and medical comorbidities, this study of over 106,000 patients demonstrated that IDA was associated with prolonged in-hospital LOS, in addition to higher rates of medical complications and healthcare expenditures following RTKA.

There are limitations associated with a retrospective database study. Due to the use of an administrative medical claims database, the validity of the study relied on the accuracy of the coding of the diagnoses and procedures, and it has been estimated that there are up to 3% of coding errors within the Medicare administrative claims database [18]. In addition, the study assessed the association between an IDA diagnosis code and outcomes following RTKA. While comorbidities were controlled for, it is possible that IDA can be a marker for another disease process or influenced from another confounding variable from poor overall health. The claims database does not record laboratory values at either pre- or postoperative time points, which, however, can be an endpoint for future prospective studies. Additionally, due to the relatively wide time period, LOS data from earlier than 2014 might not be representative of the current time point, due to decreasing in-hospital LOS secondary to healthcare reform and the introduction of rapid recovery pathways [26]. Furthermore, selection bias is commonly associated with case-control studies. While appropriate adjustments were made within the investigation by matching the study cohort to the comparison cohort by comorbidities which are commonly associated with IDA, selection bias might still be present within the investigation, which could potentially impact the results of the study. While the study used diagnostic codes which have been used in previously published arthroplasty studies, due to restrictions within the PearlDiver platform, the severity of IDA could not be assessed, as this could potentially impact the dependent variables measured in this study, and can potentially serve as the basis for future prospective investigations. Despite these limitations, there is value to the study as it utilized multivariate statistical analyses to determine the association between IDA and increased postoperative complications following RTKA, which can be used by orthopedists to guide patient care and promote further investigation into this topic.

The results of this study coincide with previously published investigations [7, 8, 27, 28]. This study found that IDA was associated with longer in-hospital LOS following RTKA compared to the control cohort. This was consistent with a single-institution, case-controlled study of 13,563 patients undergoing primary or revision TJA by Viola *et al.,* which found that patients with preoperative anemia had increased LOS [7]. In addition, a retrospective cohort study analyzing 2,394 patients undergoing elective primary TKA by Abdullah *et al.* found that preoperative anemia significantly increased LOS with increasing severity of the disease in patients with either mild anemia [adjusted OR (aOR) 1.71, *P* < 0.001] or moderate to severe anemia, (aOR 2.29, *P* < 0.001) [27]. A retrospective study by Mathew *et al.* found that patients undergoing primary TKA with IDA had longer in-hospital LOS (4-days *vs*. 3-days, *P* < 0.0001) [8]. However, limitations to the aforementioned studies include findings of the study not being generalizable to patients within the United States, not utilizing multivariate Logistic regression analyses to control for additional confounding variables, and not using a comprehensive list of diagnostic codes associated with IDA. In addition, while IDA remains the most common type of anemia, the studies mentioned did not distinguish IDA from other forms of anemia, such as anemia, from chronic diseases and other conditions, unlike the current study that only included IDA. In addition to the longer in-hospital course, the study showed IDA patients had significantly higher rates of morbidity compared to their counterparts undergoing RTKA.

IDA patients undergoing RTKA, compared to matched controls, were associated with significantly higher rates of pneumonia (OR: 6.86, *P* < 0.0001), respiratory failure (OR: 5.95, *P* < 0.0001), infections (OR: 3.22, *P* < 0.0001), and venous thromboemboli (OR: 3.22, *P* < 0.0001). While there is a lack of studies investigating pulmonary and venous thromboembolic complications following TJA in IDA patients, the study was consistent with other studies looking into postoperative complications following other surgical procedures. In a retrospective cohort analysis of 227,425 patients undergoing major non-cardiac surgery, Musallam *et al.* found that patients with anemia had higher rates of morbidities, including venous thromboemboli (OR: 1.57, *P* < 0.05) and respiratory complications (OR: 1.70, *P* < 0.05) [29]. Another retrospective study of 2,064 patients in Africa by White *et al.* found that patients with severe anemia were more likely to be admitted to the ICU postoperatively from complications including myocardial infarction, renal failure, deep vein thrombosis and pneumonia (OR: 26.58, *P* < 0.001) [30]. However, there are limitations to the above-mentioned studies as they included major non-cardiac surgeries not limited to orthopedic surgeries and did not further subdivide the study cohort to analyze any potential differences between surgery types. They also have limitations??? being generalizable to IDA patients in the United States and for not controlling for additional confounding variables through multivariate Logistic regression analysis.

Experimental evidence found that iron is essential for the normal development of the immune system [31]. In iron deficiency, the decreased function of the immune system is mediated through impaired humoral immunity, phagocytic activity, oxidative burst, and decreased interleukin-6 (IL-6) levels which can account for the increased risk of infections [31]. In addition, IDA is associated with an increased concentration of factor VIII, which is a risk factor for thrombosis [32, 33]. Anemia is also associated with a greater risk of symptomatic venous thromboembolism among acutely-ill medical patients such that patient risk can be stratified by hemoglobin levels [34]. These hematopoietic derangements could potentially explain the higher rates of infections and thromboembolic complications which were observed in the study cohort. In addition to the higher rates of complications noted in the study, IDA patients undergoing RTKA were found to have higher costs of day of surgery and EOC compared to matched controls.

The study showed IDA patients incurred significantly higher day of surgery ($16,976.01 *vs*. $14,515.81) and 90-day EOC costs ($22,548.71 *vs*. $16,819.15) when compared to patients without IDA. This was consistent with another retrospective study that reported a significant increase of $15,869 in 90-day charges for patients with concomitant anemia undergoing primary joint arthroplasty [28]. One possible explanation is that the IDA patients had significantly higher rates of medical complications following RTKA, so they incurred additional costs to treat the complications. Additional in-hospital estimated costs associated with acute renal failure treatment in the United States range from $7,469 to $33,161, depending on the severity of the illness [35]. The estimated cost to treat pneumonia ranges from $1,126.9 for emergency department/urgent care visits to $10,962.50 for hospitalizations [36]. The cost range of surgical site infections depends on the severity and location but averages to an additional cost of $5,155 [37]. The additional medical complications and their respective treatments could account for differences in cost observed.

Reducing the incidence of medical complications can have a crucial impact on reducing the associated healthcare costs. A recent meta-analysis has found that a multidisciplinary patient blood management (PBM) program can reduce perioperative complication rates and be effective in improving clinical outcomes in patients with IDA [38]. The first pillar of a PBM program is to optimize erythropoiesis by treating iron deficiency [39]. Monitoring and correcting a patient’s iron levels can be essential to improving anemia management prior to surgery [40]. Oral iron may be preoperatively given for mild-to-moderate anemia if there is sufficient time (6 to 8 weeks) and adequate tolerance to oral preparations [40]. Intravenous iron should be used in cases of moderate-to-severe IDA, short time to surgery, and for postoperative anemia management [40]. In a meta-analysis of 17 studies with 235,779 surgical patients by Althoff *et al.*, the implementation of a PBM program significantly reduced transfusion rates by 39% (*P* < 0.00001), hospital length of stay (*P* < 0.00001), total number of complications (*P* < 0.00001), and mortality rate (*P* = 0.02) [38]. Using a PBM program to manage a patients’ anemia can, in turn, reduce the overall costs following surgery.

## Conclusion

This investigation found patients who had IDA undergoing RTKA had significant increase in LOS, healthcare costs, and frequency of adverse events within 90-days following RTKA. Future studies should aim at comparing the severity and duration of IDA on outcomes following the procedure, as this could potentially impact outcomes. There are currently no specific guidelines on how to manage preoperative anemia prior to RTKA, which could potentially lower medical complications and costs. The study is useful as it can be used by orthopedic surgeons and other healthcare professionals to adequately educate these patients of the potential complications following their procedure.

## Supplementary Information

**Additional file 1.**

## Data Availability

Not applicable.

## Abbreviations

RTKA: Revision Total Knee Arthroplasty
IDA: Iron Deficiency Anemia
ECI: Elixhauser-Comorbidity Index
CCI: Charon-Comorbidity Index
OR: Odds-Ratio
95%CI: 95% Confidence Interval
ICD-9: International Classification of Disease, Ninth Revision
TJA: Total Joint Arthroplasty
TKA: Total Knee Arthroplasty
CPT: Current Procedural Terminology
IRB: Institutional Review Board
COPD: Chronic Obstructive Pulmonary Disease
LOS: In-hospital lengths of stay
