# Supplementary material for: The association of iron deficiency anemia and perioperative complications following revision total knee arthroplasty
Source: Arthroplasty. 2022 Jul 27;4:34. doi: 10.1186/s42836-022-00129-4 (PMC9327154; doi:10.1186/s42836-022-00129-4)
Supplement: Supplementary file 1 — Additional file 1. [file 42836_2022_129_MOESM1_ESM.docx]

| **Procedure &/Or Diagnosis** | **ICD-9 or CPT Code Utilized** |
| --- | --- |
| Revision Total Knee Arthroplasty | ICD-9-P-8457, ICD-9-P-8155, ICD-9-P-0080, ICD-9-P-0081, ICD-9-P-0082, ICD-9-P-0083, ICD-9-P-0084 |
| Iron Deficiency Anemia | ICD-9-D-2800, ICD-9-D-2801, ICD-9-D-2808, ICD-9-D-2809 |
| Acute Kidney Injury | ICD-9-D-5845, ICD-9-D-5846, ICD-9-D-5847, ICD-9-D-5848, ICD-9-D-5849, |
| Cerebrovascular Accidents | ICD-9-D-430, ICD-9-D-431, ICD-9-D-4320: ICD-9-D-4329, ICD-9-D-4330: ICD-9-D-43391, ICD-9-D-4340: ICD-9-D-43491, |
| Chronic Obstructive Pulmonary Disease | ICD-9-D-491.22 |
| Coronary Artery Disease | ICD-9-D-4110: ICD-9-D-4149, |
| Deep Vein Thromboses | ICD-9-D-4532, ICD-9-D-4533, ICD-9-D-4534, ICD-9-D-45382, ICD-9-D-45384, ICD-9-D-45385, ICD-9-D-45386, |
| Diabetes Mellitus | ICD-9-D-24900: ICD-9-D-25099, ICD-9-D-7902, ICD-9-D-79021, ICD-9-D-79022, ICD-9-D-79029, ICD-9-D-7915, ICD-9-D-7916, |
| Hyperlipidemia | ICD-9-D-E7841 |
| Hypertension | ICD-9-D-4010: ICD-9-D-4059, |
| Ileus Episodes | ICD-9-D-5601 |
| Myocardial Infarction | ICD-9-D-41000: ICD-9-D-41092 |
| Obesity | ICD-9-D-2780, ICD-9-D-27800, ICD-9-D-27801, ICD-9-D-27802, ICD-9-D-27803, |
| Pneumonia | ICD-9-D-4800:ICD-9-D-4809, ICD-9-D-481, ICD-9-D-4820, ICD-9-D-4821, ICD-9-D-48230, ICD-9-D-48231, ICD-9-D-48232, ICD-9-D-48239, ICD-9-D-48240, ICD-9-D-48241, ICD-9-D-48242, ICD-9-D-48249, ICD-9-D-48281, ICD-9-D-48282, ICD-9-D-48283, ICD-9-D-48284, ICD-9-D-48289, ICD-9-D-4829, ICD-9-D-4830, ICD-9-D-4831, ICD-9-D-4838, ICD-9-D-4841, ICD-9-D-4843, ICD-9-D-4845, ICD-9-D-4846, ICD-9-D-4847, ICD-9-D-4848, ICD-9-D-485, ICD-9-D-486, |
| Pulmonary Emboli | ICD-9-D-4151:ICD-9-D-4159, |
| Surgical Site Infections | ICD-9-D-99859, ICD-9-D-99851, ICD-9-P-8604, ICD-9-P-8006, CPT-10180, CPT-20005, CPT-27310, CPT-11981, CPT-29871 |
| Tobacco | ICD-9-D-3051, ICD-9-D-98984, ICD-9-D-V1582, |
| Transfusion of Blood Products | ICD-9-P-99.x |
| Urinary Tract Infections | ICD-9-D-5990 |
| Venous Thromboemboli | ICD-9-D-4532, ICD-9-D-4533, ICD-9-D-4534, ICD-9-D-45382, ICD-9-D-45384, ICD-9-D-45385, ICD-9-D-45386, ICD-9-D-4151: ICD-9-D-4159, |

Supplementary Table 1. List of International Classification of Disease or Current Procedural

Terminology Codes Utilized
